# The influence of participant characteristics on the relationship between cuff pressure and level of blood flow restriction

**DOI:** 10.1007/s00421-016-3399-6

**Published:** 2016-05-27

**Authors:** Julie E. A. Hunt, Clare Stodart, Richard A. Ferguson

**Affiliations:** School of Sport, Exercise and Health Sciences, Loughborough University, Loughborough, Leicestershire LE11 3TU UK; Faculty of Health and Medical Sciences, School of Biosciences and Medicine, University of Surrey, Guildford, GU2 7YW UK

**Keywords:** Blood flow restriction, Arterial occlusion, Training

## Abstract

**Purpose:**

Previous investigations to establish factors influencing the blood flow restriction (BFR) stimulus have determined cuff pressures required for complete arterial occlusion, which does not reflect the partial restriction prescribed for this training technique. This study aimed to establish characteristics that should be accounted for when prescribing cuff pressures required for partial BFR.

**Methods:**

Fifty participants were subjected to incremental blood flow restriction of the upper and lower limbs by proximal pneumatic cuff inflation. Popliteal and brachial artery diameter, blood velocity and blood flow was assessed with Doppler ultrasound. Height, body mass, limb circumference, muscle–bone cross-sectional area, adipose thickness (AT) and arterial blood pressure were measured and used in different models of hierarchical linear regression to predict the pressure at which 60 % BFR (partial occlusion) occurred.

**Results:**

Combined analysis revealed a difference in cuff pressures required to elicit 60 % BFR in the popliteal (111 ± 12 mmHg) and brachial arteries (101 ± 12 mmHg). MAP (*r* = 0.58) and AT (*r* = −0.45) were the largest independent determinants of lower and upper body partial occlusion pressures. However, greater variance was explained by upper and lower limb regression models composed of DBP and BMI (48 %), and arm AT and DBP (30 %), respectively.

**Conclusion:**

Limb circumference has limited impact on the cuff pressure required for partial blood flow restriction which is in contrast to its recognised relationship with complete arterial occlusion. The majority of the variance in partial occlusion pressure remains unexplained by the predictor variables assessed in the present study.

## Introduction

Blood flow restricted (BFR) exercise is a training modality generating substantial research interest. Studies have demonstrated that this training stimulus can elicit similar hypertrophic and strength gains as traditional heavy load resistance exercise (Takarada et al. 2000; Karabulut et al. 2010), improve skeletal muscle endurance (Takarada et al. 2002; Loeppky et al. 2005; Sumide et al. 2009; Kacin a and Strazar 2011) and prompt vascular remodelling (Evans et al. 2010; Hunt et al. 2013). Whilst the potential benefits are clear, many studies observe a wide range of individual differences in the acute and chronic response to BFR training. Notwithstanding the role of individual biological variability (Roth 2008), the additional variables encountered with BFR exercise may be responsible for the contradicted training outcomes.

Perhaps the most obvious consideration is the variance in the level of blood flow restriction between individuals. It is common in BFR exercise protocols for the cuff to be inflated to the same absolute occlusion pressure (mmHg) (e.g. Takarada et al. 2000; Abe et al. 2006; Madarame et al. 2008; Sakuraba and Ishikawa 2009; Evans et al. 2010; Credeur et al. 2010; Kacin a and Strazar 2011; Yasuda et al. 2012). However, a standard occlusion pressure may not reduce blood flow to the same degree in different individuals. As such, the level of oxygen delivery as well as accumulation and clearance rate of local metabolic by-products during BFR exercise are likely to be different between individuals (Takarada et al. 2000; Yasuda et al. 2008; Karabulut et al. 2011). Complete arterial occlusion causes greater ratings of perceived exertion (Sumide et al. 2009; Yasuda et al. 2009) compared to partial occlusion and limits the tolerable duration of exercise (Cook et al. 2007; Yasuda et al. 2009), reducing the effectiveness of BFR resistance training (Sumide et al. 2009; Kacin a and Strazar 2011). Indeed, achieving a high exercise volume appears crucial, as it is the potential mediating factor for skeletal muscle hypertrophy (Burd et al. 2010) and vascular remodelling (Prior et al. 2003). Therefore, efficacy of BFR resistance exercise seems to occur with the suppression of venous outflow (causing pooling of the blood) and partial but not complete reduction in arterial inflow. Moreover, occlusion protocols eliciting an approximate 60 % reduction in resting blood flow (as quantified by Takano et al. 2005) appear to be effective (Kim et al. 2009; Sumide et al. 2009; Evans et al. 2010; Karabulut et al. 2010; Patterson and Ferguson 2010; Suga et al. 2010; Cook et al. 2010; Hunt et al. 2013).

The level of BFR is influenced by the tourniquet cuff design and application method. The width of the cuff is an important variable to consider. Wide cuffs transmit a greater percentage of the applied tourniquet pressure to deeper tissues than narrow cuffs and are therefore more effective in restricting arterial blood flow at lower inflation pressures (Crenshaw et al. 1988). Indeed, Loenneke et al. (2011) found the cuff pressure required for complete arterial occlusion was significantly lower in wide (144 mmHg; 13.5 cm wide, Hokanson) compared to narrow (235 mmHg; 5 cm narrow, Kaatsu Master) cuffs prevalently used in BFR research. Wide cuffs are, therefore, recommended as they reduce the necessary pressure application for partial occlusion (Wernbom et al. 2006).

Rarely do investigations control for individual factors that influence the relative level of BFR. For the few studies that have attempted to do so, the most common method is to adjust the cuff pressure according to individual’s systolic blood pressure (~130 % SBP) (Takano et al. 2005; Karabulut et al. 2006; Clark et al. 2011; Cook et al. 2010). Whilst this seems to work successfully in training studies using narrow cuffs no equivalent calculation for use with wider cuffs has been provided. Partial occlusion (~60 %) is likely to occur, using wider cuffs, at much lower pressures than 130 % systolic blood pressure (SBP). In fact, findings by Loenneke et al. (2011) suggest wide cuff inflation at 130 % SBP (130 % of ~120 = 156 mmHg) would exceed the necessary pressure for complete arterial occlusion (144 mmHg) in healthy individuals. Even so there is debate whether SBP should even be considered since studies report a moderate (*r* = 0.56, Younger et al. 2004) if not absent [*r* = 0.05 (Crenshaw et al. 1988; Loenneke et al. 2011)] relationship between SBP and limb occlusion pressure. This suggests that basing cuff pressure on SBP alone may not lead to optimal BFR and further variables should be investigated.

The relative degree of BFR may be subject to the amount of the tissue surrounding the blood vessel as this influences the pressure exerted on the vasculature. In fact, limb circumference explained most of the variance in the cuff pressure required to occlude arterial flow (Loenneke et al. 2011) and may influence the fatigue response and degree of muscle hypoxia during BFR exercise (Yasuda et al. 2008). On this basis, a different cuff pressure would be required to elicit optimal BFR on upper and lower limbs. Applying just 50 mmHg on the upper arm increases electromyographic activity during elbow flexion exercise (Takarada et al. 2000) suggesting blood flow may be restricted at lower pressures than typically observed in lower limb exercise models. Yet to our knowledge, the difference in blood flow during upper and lower limb occlusion using wide cuffs has not been identified. Restrictive pressures based on limb size are advised (Fahs et al. 2012), but without reporting absolute values there is limited means of practical application.

Less is understood about the impact of tissue type (muscle or fat) on the level of BFR. It is likely the degree of intramuscular pressure depends on the architecture features of the muscle. Hypertrophied muscles have a greater thickness and volume, and subsequently blood flow occlusion may occur at relatively lower pressures (Wernbom et al. 2006). Despite this, fat as opposed to muscle cross-sectional area (CSA) explained most of the variance in the cuff pressure required to completely occlude arterial flow (Loenneke et al. 2011). It is not known how limb composition influences BFR over the range of venous occlusion pressures typically employed during training.

Previous investigations trying to establish the factors (blood pressure, limb size and composition) influencing the BFR stimulus have determined the external cuff pressure required for complete arterial occlusion (100 % BFR) (Loenneke et al. 2011), which does not reflect the partial restriction prescribed for this training technique. Assumptions of a linear relationship between external cuff pressure and the level of BFR cannot be made. Indeed, Laurentino et al. (2012) reported that 50 % BFR (relative to resting blood flow) was achieved at 80 % of the pressure required to occlude arterial flow, suggesting a non-linear relationship between these factors.

Therefore, the aim of this study was to establish whether subject characteristics (i.e., arterial blood pressure, limb size and composition) should be accounted for when prescribing the cuff pressure required for BFR. The latter was estimated by determining the relationship between cuff pressure and the level of blood flow restriction for each individual which was achieved by measuring the changes in conduit artery diameter, blood velocity and blood flow in response to proximal cuff inflation over a range of external pressures.

## Methods

### Participants

Fifty participants (25 male, 25 female; age; 21 ± 3 years, height; 1.76 ± 0.10 m, body mass; 69.7 ± 12.1 kg) volunteered to take part in the investigation. All were fully informed of the purposes, risks and discomforts associated with the experiment before providing written consent. The study conformed to guidelines outlined in the Declaration of Helsinki and was approved by Loughborough University Ethics Advisory Committee.

### Experimental protocol

All tests were conducted 4 h post-prandial in a quiet, temperature-controlled room (24 ± 1 °C). Participants abstained from exercise, alcohol and tobacco for 24 h prior to the experimental test. Upon arrival into the laboratory, participant’s height and body mass were measured using a stadiometer and manual scales. Anthropometric assessments of the right thigh and arm were made, followed by B-mode ultrasound measures of anterior and posterior subcutaneous adipose tissue thickness. Participants then rested supine for 20 min prior to arterial blood pressure measurement and vascular assessments. Doppler ultrasound was used to image the popliteal and brachial artery in response to incremental blood flow restriction induced by proximal pneumatic cuff inflation.

### Anthropometry, adipose thickness and estimated muscle–bone CSA

Height (m) and body mass (kg) were measured with a stadiometer and scales, respectively. Body mass index (BMI) was calculated as kg/m^2^. Thigh circumference was measured horizontal to the long axis of the femur at the mid-point between the inguinal fold and the anterior aspect of the patella. The horizontal (circumferential) line was marked and extended to intercept with the vertical mid-line of the anterior and posterior surface of the thigh. Subcutaneous adipose tissue thickness overlying the quadriceps and hamstrings were measured at these sites. Arm circumference was measured horizontal to the long axis of the humerus at the mid-acroniale-radiale distance. The horizontal (circumferential) line was marked and extended to intercept with the vertical mid-line of the anterior and posterior surface of the arm. Subcutaneous adipose tissue thickness overlying the biceps and triceps were measured at these sites.

Orientated in the sagittal plane, the centre of the ultrasound transducer was aligned perpendicular to the horizontal (circumferential) line, on the anterior and posterior surface of the limb. Subcutaneous adipose tissue was imaged at depth of 2–5 cm, depending on the site and individual. Minimal force was applied through the transducer to avoid compression of the adipose layer. Two ultrasound B-mode images (Toshiba Powervision 6000 with a multi-frequency linear array transducer; 7–11 MHz) were systematically acquired at each site (quadriceps, hamstrings, biceps and triceps) and later analysed using Image J software. On each image, subcutaneous fat thickness was measured between skin–fat and fat–muscle interfaces at four sites and the average of eight values recorded. Limb adipose thickness (AT) was determined by averaging values attained at anterior and posterior sites [thigh AT = (quadriceps AT + hamstring AT)/2; arm AT = (brachial AT + triceps AT)/2]. Muscle–bone CSA was estimated using the formula *π*[(*r* − AT)^2^] where r is the radius of the limb calculated as circumference/2*π* and AT is ultrasound measured adipose thickness (Bemben et al. 2005).

### Blood pressure

Systolic (SBP) and diastolic (DBP) blood pressures were measured from the brachial artery, following 5-min supine rest, using an automated blood pressure cuff (Omron M5-1 Digital BP monitor, Omron Healthcare, The Netherlands). Blood pressure was taken in triplicate and the closest two values averaged for analysis. Mean arterial pressure (MAP) was calculated as DBP + (SBP − DBP)/3 in accordance with Levick (2011).

### Ultrasound measures of blood flow restriction

*Popliteal artery* Participants lay in a prone position for 20 min before the test protocol commenced. A 13 cm wide pneumatic cuff (SC12L™ segmental pressure cuff, Hokanson, WA) was positioned on the right thigh overlying marks used for the prior anthropometric assessment. Popliteal artery diameter and velocity were measured using duplex ultrasound behind the popliteal fossa at depths of 3–4 cm (11–9 MHz). All images of the popliteal artery were acquired proximal to the branching of the tibial artery; however, distance variation between participants was required to ensure the highest possible image quality. Baseline measures of the artery were recorded for 30 cardiac cycles before commencing the inflation protocol. The thigh cuff was initially inflated (E20 Rapid Cuff Inflator and AG101 Cuff Inflator Air Source, Hokanson, WA) to 90 mmHg for 150 s and then deflated for 180 s. Ultrasound images of the popliteal were recorded for the final 30 s of each inflation step (after 2 min of inflation) to ensure attainment of steady state flow (Takarada et al. 2000). This procedure was repeated and cuff pressure was increased incrementally by 10 mmHg (150 s inflation, 180 s deflation) until 150 mmHg was reached or until the popliteal flow was no longer detected.

*Brachial artery* Participants moved to a supine position with their right arm extended and immobilised at an angle approximately 80° from the torso. An 11 cm wide pneumatic cuff (SC10 segmental pressure cuff, Hokanson, WA) was positioned on the upper arm overlying marks of prior anthropometric assessment. Brachial artery diameter and velocity were measured using duplex ultrasound directly downstream of the cuff >3 cm proximal of the olecranon process. With the starting pressure of 60 mmHg the same inflation/deflation procedure for ultrasound image acquisition was used as described for the popliteal artery.

*Analysis of blood flow restriction* Popliteal and brachial artery diameter and flow velocity during each pressure step were analysed with a custom-designed, edge detection and wall tracking software (Vascular Research Tools 5, Medical Imaging Applications, LLC, Coralville, Iowa). Media-to-media diastolic diameter was measured within a specified region of interest on B-mode images. The Doppler flow velocity spectrum was traced and TAMV (cm/s) computed. Synchronised diameter and velocity data, sampled at 20 Hz, enabled calculation of blood flow and shear rate. Resting diastolic diameter (mm) was averaged over 30 cardiac cycles. Blood flow (ml/min) was calculated as (TAMV × *πr*^2^) × 60, where *r* is the radius of the artery lumen. Resting blood flow was averaged over 20 cardiac cycles. Shear rate was derived from Poiseuillies law and calculated accordingly as (4 × TAMV)/diameter. The day-to-day reproducibility of resting brachial artery measurements were; diameter (0.2 %), velocity (16 %) and blood flow (16 %). The day-to-day reproducibility of resting popliteal artery measurements were; diameter (0.5 %), velocity (16 %) and blood flow (17 %).

### Statistical analysis

Data was analysed using SPSS statistical package (IBM SPSS Statistics 20). A Shapiro–Wilk test was used to confirm normal distribution and a Mauchley test of sphericity to verify homogeneity of variance. An independent *t* test determined differences in baseline conduit artery variables (diameter, blood velocity and blood flow) between sexes. A two-way mixed measures ANOVA was used to evaluate the conduit artery response (diameter, blood velocity and blood flow) to incremental pressures (popliteal 90–150 mmHg, brachial 60–120 mmHg) in males and females. This was followed by one-way repeated-measures ANOVA to confirm change with incremental pressures in males and females separately. Bonferroni corrected post hoc *t* tests were then used to locate significance. Blood flow at each cuff pressure was expressed relative to baseline (without cuff) to quantify the level of restriction. Individual data were then fitted with a second order polynomial equation for the prediction of cuff pressure (mmHg) at 60 % BFR. Pearson’s correlation was then used to determine the relationship between dependent (cuff pressure at 60 % BFR) and predictor variables (anthropometry, adipose thickness, muscle CSA and blood pressure). This provided an order for entering variables into the hierarchical linear regression models. Different models of hierarchical linear regression were used to predict the pressure at which 60 % BFR occurred in both the popliteal and brachial artery. Models consisted of 2–3 blocks. Pearson correlation, SEE and the change in the *F* value was assessed as each individual variable was added to the overall model. Only a single variable for blood pressure (SBP, DBP, MAP), anthropometry (body mass, BMI) and limb specific morphology (circumference, adipose thickness and muscle CSA) was entered in each model to avoid multi-colinearity. There was no concern regarding multi-colinearity among predictor variables in the hierarchical linear regression models [variance inflation factor (VIF) <2.7].

## Results

Ultrasound images were of insufficient quality in three participants preventing quantification of blood flow restriction, and were therefore excluded from the results. Whole group subject characteristics are presented in Table 1 (*n* = 47; 24 males, 23 females).

**Table 1:** Participant characteristics

Variable	Males (*n* = 24)	Females (*n* = 23)	Combined (*n* = 47)	Minimum (*n* = 47)	Maximum (*n* = 47)
Age (years)	22 ± 3	21 ± 2	21 ± 3	18	32
Height (m)	1.81 ± 0.07	1.67 ± 0.07	1.76 ± 0.10	1.52	1.95
Mass (kg)	76.5 ± 10.1	60.8 ± 8.4	69.7 ± 12.1	46.1	94.7
BMI (kg/m^2^)	23.1 ± 2.4	21.7 ± 2.7	22.5 ± 2.6	18.2	29.3
SBP (mmHg)	132 ± 11	112 ± 7	124 ± 14	96	163
DBP (mmHg)	75 ± 7	69 ± 5	72 ± 7	61	88
MAP (mmHg)	94 ± 7	84 ± 5	89 ± 8	73	106
Thigh circumference (cm)	57.9 ± 3.9	55.5 ± 4.1	57.0 ± 4.1	50.2	66.7
Thigh AT (mm)	9.8 ± 3.9	16.5 ± 4.5	12.8 ± 5.3	3.2	27.0
Thigh muscle–bone CSA (cm^2^)	214 ± 30	163 ± 22	193 ± 37	131	275
Arm circumference (cm)	33.4 ± 30	29.5 ± 2.8	31.6 ± 3.5	24.6	38.7
Arm AT (mm)	6.4 ± 3.3	10.3 ± 4.0	8.2 ± 4.1	1.9	22.1
Arm muscle–bone CSA (cm^2^)	70 ± 14	43 ± 9	57 ± 18	33	102

Values are mean ± SD

*BMI* Body mass index, *SBP* systolic blood pressure, *DBP* diastolic blood pressure, *MAP* mean arterial pressure, *AT* adipose thickness

### Conduit artery response to incremental external cuff pressure

**Fig. 1:**
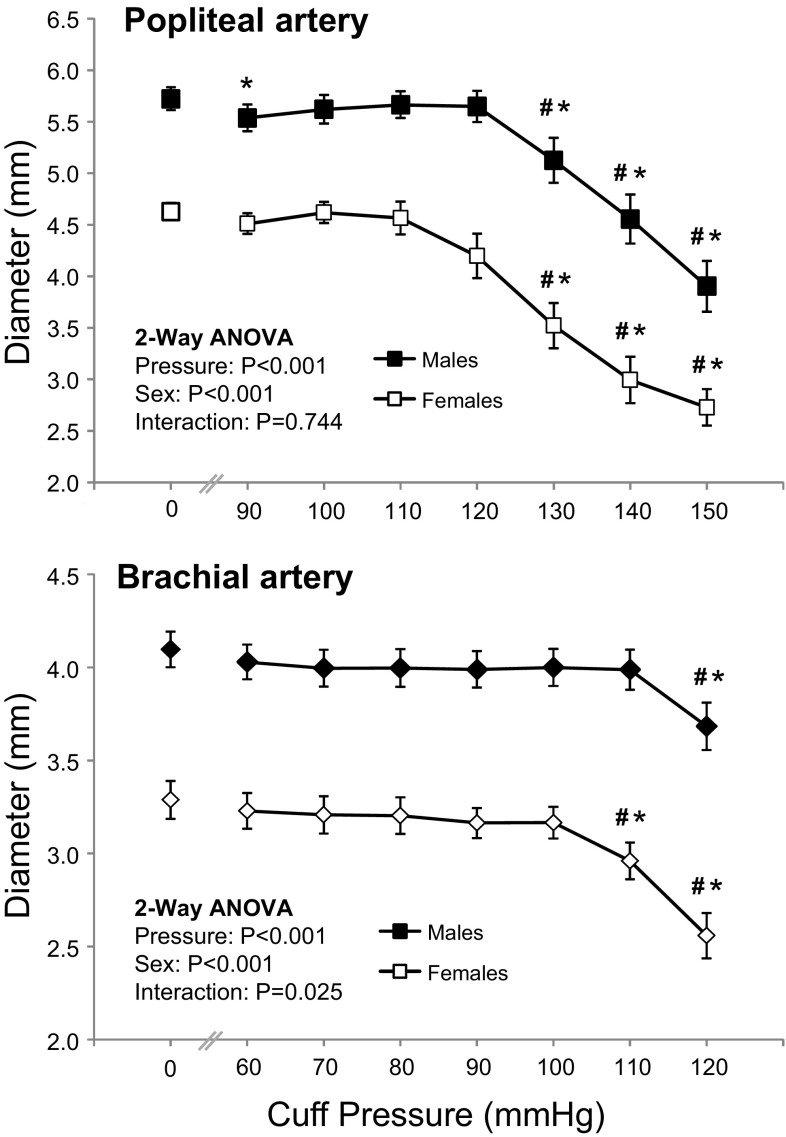
Diameter of the popliteal and brachial arteries at different external cuff pressures in males and females. Values are mean ± SEM. *Significantly different to resting value (*P* < 0.05, Bonferroni *t* test). ^#^Significantly different to previous cuff pressure (*P* < 0.05, Bonferroni *t* test)

Female participants had smaller (*P* < 0.001) popliteal (4.61 ± 0.41 vs. 5.72 ± 0.60 mm) and brachial (3.29 ± 0.48 vs. 4.10 ± 0.52 mm) arteries than males. Popliteal and brachial artery diameters (Fig. 1) began to decrease (2-way ANOVA main effect for cuff pressure, *P* < 0.001) with external pressures of 130 and 110 mmHg (Bonferroni post hoc, *P* = 0.015 and *P* < 0.001, respectively). The artery diameter response to incremental pressure differed between the sexes in the brachial but not the popliteal artery (2-way ANOVA, pressure by sex interaction; *P* = 0.025 and *P* = 0.744). Contrasts revealed that the diameter of the brachial artery decreased at a lower pressure in the females (110 mmHg) compared to the males (120 mmHg) (*P* = 0.023).

**Fig. 2:**
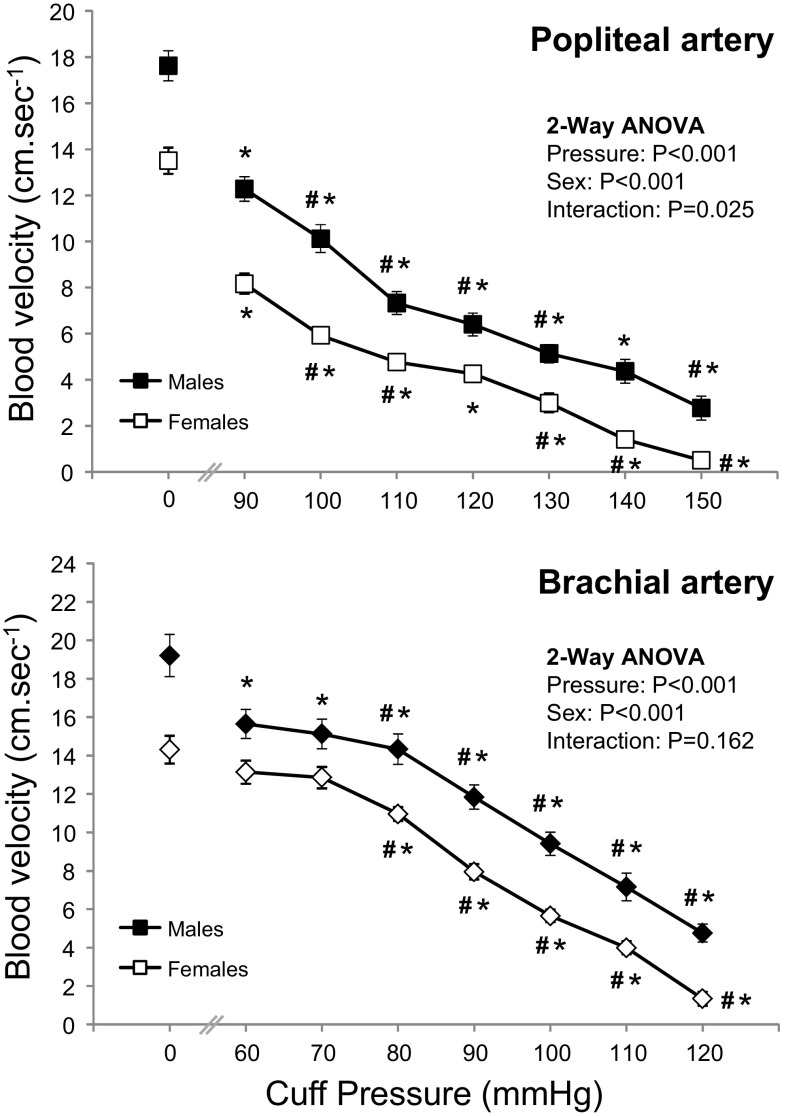
Blood velocity in the popliteal and brachial arteries at different external cuff pressures in males and females. Values are mean ± SEM. *Significantly different to resting value (*P* < 0.05, Bonferroni *t* test). ^#^Significantly different to previous cuff pressure (*P* < 0.05, Bonferroni *t* test)

Male participants demonstrated higher baseline blood velocity in the popliteal (17.6 ± 3.5 vs. 13.7 ± 2.6 cm s^−1^, *P* < 0.001) and brachial (19.2 ± 5.9 vs. 14.3 ± 3.4 cm s^−1^, *P* = 0.001) arteries compared to females. Blood velocity (Fig. 2) decreased in popliteal and brachial arteries at initial cuff inflation (vs. baseline) and with each pressure increment (2-way ANOVA main effect for cuff pressure *P* < 0.001; Bonferroni post hoc *P* < 0.05). The blood velocity response to incremental pressure (Fig. 2) differed between the sexes in the popliteal but not the brachial artery (2-way ANOVA, pressure by sex interaction; *P* = 0.025 and *P* = 0.162).

**Fig. 3:**
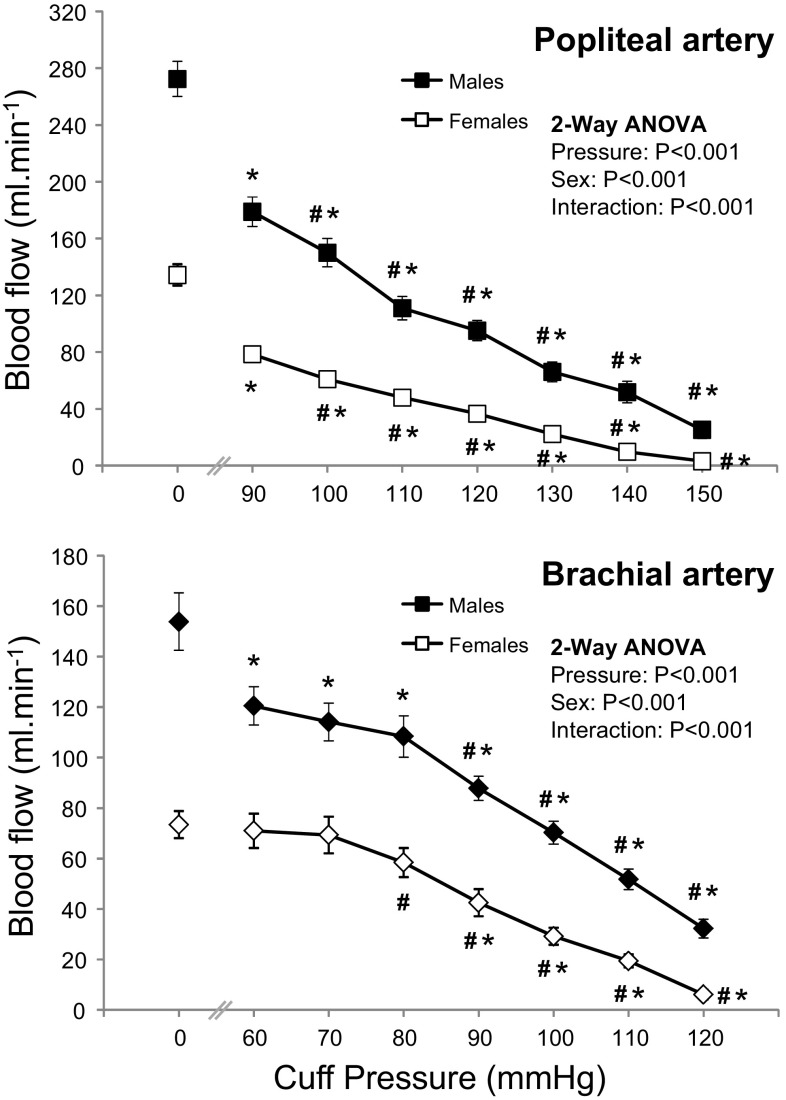
Blood flow in the popliteal and brachial arteries at different external cuff pressures in males and females. Values are mean ± SEM. *Significantly different to resting value (*P* < 0.05, Bonferroni *t* test). ^#^Significantly different to previous cuff pressure (*P* < 0.05, Bonferroni *t* test)

Baseline blood flow was lower (*P* < 0.001) in the popliteal (272 ± 67 vs. 136 ± 37 ml min^−1^) and brachial (154 ± 61 vs. 73 ± 25 ml min^−1^) arteries in females compared to males. Blood flow (Fig. 3) decreased in the popliteal and brachial arteries at initial cuff inflation (vs. baseline) and with each pressure increment (2-way ANOVA main effect for cuff pressure *P* < 0.001; Bonferroni post hoc *P* < 0.05). This response differed between the sexes (2-way ANOVA, pressure by sex interaction; *P* < 0.001). Contrasts revealed that males experienced a larger decrease in popliteal and brachial blood flow with initial cuff inflation at 90 mmHg (*P* = 0.004) and 60 mmHg (*P* = 0.008), respectively.

### External cuff pressure and the level of blood flow restriction

**Fig. 4:**
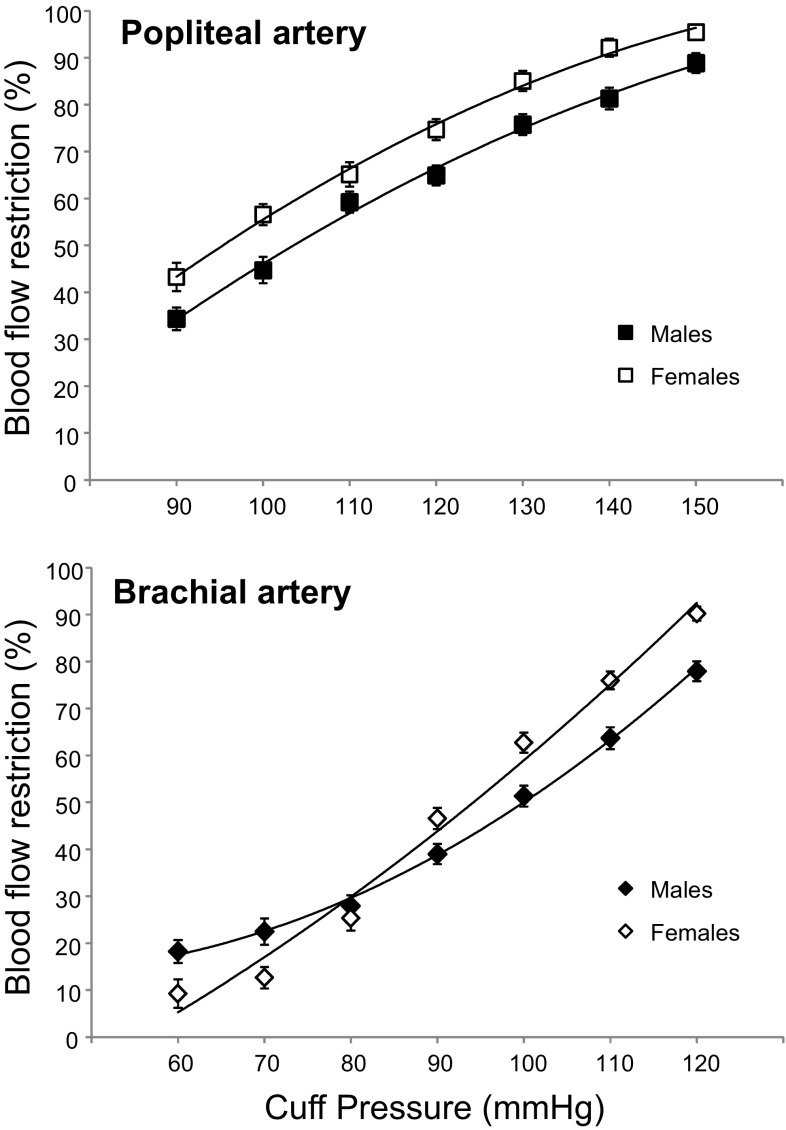
Blood flow restriction (% of resting blood flow) in the popliteal and brachial arteries at different external cuff pressure in males and females. Values are means ± 95 % confidence intervals

Individual participant blood flow restriction data was fitted with a second order polynomial equation for the prediction of cuff pressure at 60 % BFR (Fig. 4). Combined analysis revealed a significant difference in the cuff pressure required to elicit 60 % BFR in the popliteal (111 ± 12 mmHg) and brachial arteries (101 ± 12 mmHg, *P* = 0.0002). Partial BFR (60 %) was achieved in the popliteal artery at different cuff pressures for males (114 ± 15 mmHg) and females (105 ± 10 mmHg) (*P* = 0.03). In contrast, there was no difference in the pressure required to elicit 60 % BFR in the brachial artery between males (102 ± 18 mmHg) and females (100 ± 14 mmHg) (*P* = 0.67). No relationship was observed between thigh and arm cuff pressures (*r* = 0.02). Correlations between subject variables and the external cuff pressure required for 60 % BFR are presented in Table 2.

**Table 2:** Pearson correlations between subject characteristics and the external cuff pressure required for 60 % blood flow restriction in the popliteal and brachial arteries

Variable	Popliteal	Brachial
BMI (kg/m^2^)	0.44*	−0.26*
SBP (mmHg)	0.49*	0.27*
DBP (mmHg)	0.57*	0.39*
MAP (mmHg)	0.58*	0.38*
Thigh circumference (cm)	0.34*	
Thigh AT (mm)	−0.03*	
Thigh muscle–bone CSA (cm^2^)	0.31*	
Arm circumference (cm)		−0.23*
Arm AT (mm)		−0.45*
Arm muscle–bone CSA (cm^2^)		0.07*

*BMI* body mass index, *SBP* systolic blood pressure, *DBP* diastolic blood pressure, *MAP* mean arterial pressure, *AT* adipose thickness

#### Popliteal artery models

Variables were entered into the first hierarchical regression models for the popliteal in order of correlation strength. MAP had the strongest relationship with cuff pressure required for 60 % BFR [*r* = 0.58, Table 2 and block 1 of model 1 (Table 3A)] independently explaining 34 % of the variance. Entry of BMI into the regression model [block 2 of model 1 (Table 3A)] explained an additional 10 % variance (total *r*^2^ = 45 %) in the cuff pressure required for 60 % BFR, but adding thigh circumference (block 3 of model 1) did not (Sig *F* change = 0.457, Table 3A). Block 2 of model 2 (Table 3B), composed of DBP and BMI, explained the most variance (*r*^2^ = 48 %) in the cuff pressure required for 60 % BFR. The addition of thigh circumference did not explain any additional variance (Sig *F* change = 0.936). Standardised betas and part correlation coefficients indicated that DBP explained most variance from each individual block. A reduced SEE indicates an increased accuracy of prediction with model 2 compared to model 1 (9.06 vs. 8.83, respectively). To exclude potential error associated with BMI in certain populations (athletes), a third model (Table 3C) was composed of DBP and thigh circumference, which explained 41 % of the variance in the cuff pressure required for 60 % BFR.

**Table 3:** Stepwise multiple linear regression models for popliteal BFR

	*B*	SE *B*	Stand *β*	*R*	SEE	Sig *F* change
(A) Model 1^a^						
Block 1						
Constant	34.83	15.87				
MAP	0.85	0.18	0.58*	0.58	9.75	0.001
Block 2						
Constant	11.02	16.99				
MAP	0.74	0.17	0.51*			
BMI	1.49	0.53	0.33*	0.67	9.06	0.007
Block 3						
Constant	21.48	22.04				
MAP	0.76	0.17	0.52*			
BMI	1.99	0.85	0.44*			
Thigh circumference	−0.41	0.54	−0.14	0.67	9.11	0.457
(B) Model 2^b^						
Block 1						
Constant	39.62	15.58				
DBP	0.99	0.21	0.57*	0.57	9.89	0.001
Block 2						
Constant	4.65	17.07				
DBP	0.92	0.19	0.53*			
BMI	1.78	0.50	0.39*	0.69	8.83	0.001
Block 3						
Constant	5.88	23.16				
DBP	0.92	0.19	0.53*			
BMI	1.83	0.84	0.40*			
Thigh circumference	−0.04	0.53	−0.15	0.69	8.93	0.936
(C) Model 3^c^						
Block 1						
Constant	39.63	15.59				
DBP	0.99	0.21	0.57*	0.57	9.90	0.001
Block 2						
Constant	−7.71	23.24				
DBP	0.95	0.20	0.55*			
Thigh circumference	0.87	0.33	0.30*	0.64	9.31	0.012

^a^ *R* ^2^ = 0.34 Block 1, ∆*R* ^2^ = 0.102 Block 2, ∆*R* ^2^ = 0.00 Block 3. * *P* < 0.05

^b^ *R* ^2^ = 0.32 Block 1, ∆*R* ^2^ = 0.092 Block 2 (*P* < 0.05), ∆*R* ^2^ = 0.00 Block 3 (*P* = 0.937). * *P* < 0.05

^c^ *R* ^2^ = 0.32 Block 1, ∆*R* ^2^ = 0.092. * *P* < 0.05

#### Brachial artery models

Variables were entered into a hierarchical regression model for the brachial in order of correlation strength. Arm AT had the strongest relationship with cuff pressure required for 60 % BFR (*r* = −0.45, Table 2) independently explaining 20 % of the variance. Entry of DBP into the regression model [block 2 of model 1 (Table 4A)] explained an additional 9.6 % variance (total *r*^2^ = 30 %) in the cuff pressure required for 60 % BFR. Although MAP was an independent predicting variable (*r* = 0.38, Table 2) entry into the model was excluded to avoid multi-colinearity. No correlation was observed between further variables (body mass, BMI, SBP, arm circumference, muscle–bone CSA) and the cuff pressure required for 60 % BFR.

**Table 4:** Stepwise multiple linear regression model for brachial BFR

(A) Model 1	*B*	SE *B*	Stand *β*	*R*	SEE	Sig *F* change
Block 1						
Constant	108.86	2.64				
Arm AT	−0.967	0.28	−0.45	0.45	7.97	0.001
Block 2						
Constant	77.49	13.01				
Arm AT	−0.83	0.27	−.39			
DBP	0.42	0.17	0.32	0.55	7.56	0.018

*R* ^2^ = 0.206 Block 1, ∆*R* ^2^ = 0.096 Block 2, * *P* < 0.05

### Regression equations for prediction of blood flow restriction

#### Popliteal artery

The regression equations generated from model 2 and 3 were compared using mean values (Table 5). The error in prediction, as indicated by 95 % confidence intervals, was smaller using model 2 with DBP and BMI as predictor variables. With a DBP of 72 mmHg and BMI of 22.5 kg/m^2^, the predicted cuff pressure required for 60 % BFR was between 103 and 119 mmHg with approximately 68 % accuracy or 94–128 mmHg with approximately 95 % accuracy.

**Table 5:** Regression equations for popliteal and brachial artery BFR models

Popliteal artery	Model 2	Model 3
Regression equation	0.92(DBP) + 1.78(BMI) + 4.65 (±8.83)	0.95(DBP) + 0.87(ThighCir) − 7.71 (±9.31)
Pressure at 60 % BFR (mmHg)	111	110
95 % CI (mmHg)	94–128	92–128

Brachial artery	Model 1
Regression equation	0.42(DBP) − 0.83(AT) + 77.49 (±7.56)
Pressure at 60 % BFR (mmHg)	101
95 % CI (mmHg)	86–116

*BFR* blood flow restriction, *CI* confidence intervals, *BMI* body mass index, *DBP* diastolic blood pressure, *ThighCir* thigh circumference, *AT* adipose thickness

#### Brachial artery

Mean values for arm AT (8.1 mm) and DBP (72 mmHg) were entered into the regression equation generated from model 1 (Table 5). The predicted cuff pressure required for 60 % BFR was between 93 and 109 mmHg with approximately 68 % accuracy or 86–116 mmHg with approximately 95 % accuracy.

## Discussion

This study has demonstrated between-subject variation in the level of blood flow restriction at different external cuff pressures applied to upper and lower limbs. With wide cuff (13 cm) application the external pressure required to illicit 60 % BFR in the popliteal was 111 ± 12 mmHg and was greater than in the brachial at 101 ± 12 mmHg. MAP and AT were the largest independent determinants of lower and upper body partial occlusion (60 % BFR) pressures, respectively. Despite entering further variables into an upper (DBP and BMI) and lower (DBP and AT) limb regression model, the majority of variance remains unexplained.

Efficacy in BFR exercise is achieved by balancing the level of blood flow restriction, with an appropriate level of muscle activation and fatigue (contractile/metabolic impairment), so as to ensure an appropriate training load (total work done) (Yasuda et al. 2008; Fahs et al. 2012). The level of BFR will influence hemodynamic signals, oxygen and nutrient delivery, and the accumulation and clearance rate of local metabolic by-products during exercise (Takarada et al. 2000; Karabulut et al. 2011). This is likely to have implications on skeletal muscle and vascular adaptations to BFR training, since muscle mass increases in proportion to metabolic stress under ischemic conditions (Takada et al. 2012), while conduit and resistance vessel adaptation is related to the magnitude and pattern of shear stress (Green et al. 2011), and capillary growth is proportional to metabolic activity (Adair and Montani 2010). Ensuring an equal and appropriate level of BFR between participants is therefore important to achieve consistent gains from exercise training.

The inflation pressure required to elicit a 60 % decrease in blood flow to the lower and upper extremities was investigated using Doppler ultrasound to monitor blood flow in the brachial and popliteal arteries, respectively. The decision to establish a 60 % reduction in resting blood flow was based on the hemodynamic profile of narrow cuff inflation protocols, which have been shown to be effective in BFR training studies (Takano et al. 2005). Lower restrictive cuff pressures are generally utilised for upper body BFR exercise due to the smaller limb size (Fahs et al. 2012). In agreement, it was observed that the cuff pressure required to illicit 60 % BFR was lower in the brachial versus the popliteal, but only by 10 mmHg. This corresponds with recommendations for prescribing narrow cuff restrictive pressures at 120 vs. 130 % of SBP on the upper vs. lower limbs, respectively (Takano et al. 2005; Yasuda et al. 2008). However, the findings equate to 81 and 90 % SBP (based on participants average SBP, Table 1) in the present study, using wide cuffs on the upper and lower limbs. Nevertheless, the poor relationship between SBP and pressure at 60 % BFR in the popliteal (*r* = 0.49) and brachial (*r* = 0.27) arteries suggests restrictive cuff pressures should not be based on SBP alone. The restriction of brachial artery blood flow at low–moderate external cuff pressures (60–100 mmHg) was less than anticipated. Previous investigations have shown increased electromyographic activity during elbow flexion exercise at cuff pressures as low as 50 mmHg (Takarada et al. 2000). In the absence of ischemia the suppressed clearance of metabolites due to venous occlusion may have increased voluntary muscle activation. This highlights the importance of considering both venous outflow and arterial inflow when quantifying the hemodynamic response to BFR exercise.

The 60 % reduction in popliteal blood flow was achieved at lower external cuff pressures in females (105 ± 10 mmHg) compared to males (114 ± 15 mmHg). However, there was no difference in the pressure at 60 % BFR in the brachial artery between sexes (100 ± 14 mmHg vs. 102 ± 18 mmHg). This difference in responses between the popliteal and brachial artery is unlikely to be due to a disparity in limb size, since circumference of the arm and thigh are proportionately larger in males versus females. An alternative is that findings reflect differences between sexes in local vascular function. For example, Nishiyama et al. (2008) noted that women have similar vascular function to men in the upper extremities but appear to have impaired vascular function, when normalised for shear rate, in the lower extremities. Heterogeneity of endothelial function throughout the arterial tree may account for the absent relationship between the cuff pressure at 60 % BFR in the brachial versus the popliteal artery.

Stepwise regression models were used to determine what subject characteristics should be accounted for when prescribing the restriction cuff pressure for lower and upper limb BFR training. Although MAP had the strongest independent relationship with pressure at 60 % BFR in the popliteal artery, more variance (48 %) was explained by entering DBP alongside BMI in the regression model. Accounting for thigh circumference and DBP, in accordance with Loenneke et al. (2011), resulted in greater error in the prediction of the pressure required for 60 % popliteal BFR. Conversely, AT had the strongest independent relationship with pressure at 60 % BFR in the brachial artery, with DBP explaining additional variance (total 30 %). In contrast to previous conceptions, arm circumference did not influence the cuff pressure required for 60 % BFR in the brachial artery and was therefore not included in the regression model.

It was found that SBP, though often used to determine the restrictive cuff pressure during BFR exercise, did not explain additional variance in either lower or upper body regression models. This is in agreement with others who report a weak correlation between SBP and arterial occlusion pressure in normotensive adults (Moore et al. 1987; Crenshaw et al. 1988). In contrast, DBP appears a significant predictor of partial occlusion pressures, corresponding with its association with peripheral resistance (Levick 2011).

In contrast to previous investigations a weak (*r* = 0.34) and even absent (*r* = −0.23, *P* > 0.05) relationship between limb circumference and partial occlusion pressure in the popliteal and brachial arteries was observed. This was unexpected since the percentage of external cuff pressure reflected in the underlying tissue, and therefore vasculature, is inversely related to the circumference of the limb (Shaw and Murray 1982). However, Crenshaw et al. (1988) did note arterial occlusion pressure was much more dependent on thigh circumference when using narrow as opposed to wide cuffs, as used in the present investigation. Nevertheless, our regressions may be constrained by the narrow variance in limb circumference (thigh 50–67 cm; arm 25–39 cm). Loenneke et al. (2011) reported a larger range in this predictor variable (thigh circumference 56-78 cm) and, perhaps as a consequence, a stronger correlation between thigh circumference and arterial occlusion pressure. Alternatively, limb circumference may not influence partial (60 % BFR) as opposed to complete arterial occlusion measured previously (Loenneke et al. 2011). Other factors such as local vascular function may play a more predominant role in partial occlusion pressures.

The effect of limb composition on the level of blood flow restriction has been postulated (Karabulut et al. 2011). Tissue oxygenation during low level cuff inflation was negatively correlated with leg lean body mass (Karabulut et al. 2011) suggesting muscle may transmit more pressure on the underlying vasculature, while others have noted a positive relationship between muscle CSA and arterial occlusion pressure, suggesting otherwise (Loenneke et al. 2011). The present study finds a negative relationship between adipose tissue thickness and pressure at 60 % BFR in the brachial artery. Further research regarding the effect of tissue composition on the level of blood flow restriction using more sensitive measures of fat and muscle mass is warranted.

The regression models formed in the present investigation account for 48 and 30 % of the variance in lower and upper limb partial occlusion, therefore a large percentage remains unexplained. Heterogeneity in conduit artery responses to cuff inflation among healthy subjects (Harrison et al. 2011; Humphreys et al. 2014) and between arterial beds (Weissgerber et al. 2010) has been described previously. Therefore, the variance observed may be due to individual differences in vessel characteristics, such as basal tone (Humphreys et al. 2014), arterial stiffness (Harrison et al. 2011; Miyachi 2012) and endothelial function (Dawson et al. 2012). Our utilisation of duplex-Doppler ultrasound, as opposed to pulse-Doppler, allowed for insightful measures of conduit artery diameter and blood flow velocity in response to proximal cuff inflation. Conduit artery diameter was maintained at lower cuff pressures (<110 mmHg) before rapidly decreasing. This response is delayed in males, which is consistent with evidence of a more pronounced low-flow-mediated vasoconstriction in women (Levenson et al. 2001). In contrast to diameter, we report a linear decline in blood velocity with increased external cuff pressure.

An important observation is that the conduit artery does not constrict to attain a 60 % reduction in blood flow. The latter is instead due to a marked decrease in blood flow velocity (time average mean velocity). Constriction of the artery in response to low-flow is mediated by endothelin-1 (Spieker et al. 2003) or through inhibiting endothelial-derived hyperpolarising factor (EDHF) and cyclooxygenase products (e.g., prostaglandins) (Gori et al. 2008). Repeated exposure to such stimuli could promote endothelial dysfunction. BFR training studies applying higher external cuff pressures should consider the level of artery constriction and potential for functional maladaptation of the vasculature.

We acknowledge the limitations of the present study. The sample size is relatively small compared to other similar studies (e.g., Loenneke et al. 2011). However, it should be appreciated that the degree of additional information from the duplex-Doppler ultrasound measurements (artery diameter, blood velocity, blood flow etc.) far exceeds that provided by the relatively simple measure of arterial occlusion pressure via pulse-Doppler. Coupled with the significant level of post-experimental processing the duplex-Doppler ultrasound signals require we believe justifies the final sample size. Furthermore, the sample had a relatively small range in BMI (no greater than 29.3 kg/m^2^) and thigh circumference (no greater than 66.7 cm) therefore these results may not be generalizable to obese individuals (or those with limb circumferences outside the range in this study). Participants were resting in a supine position during blood flow measures, while BFR protocols involve exercise and are typically performed in the seated or standing position. A similar relationship between cuff pressure and blood flow would be expected during exercise; however, the cuff pressure required to result in a given level of blood flow restriction would likely be elevated due to the activated skeletal muscle pump and increased BP in response to postural changes and the pressor response of exercise.

In conclusion, this study used regression models to determine the impact of subject characteristics (blood pressure, limb size and composition) on the external pressure (using a 13 cm wide cuff) required to elicit 60 % reduction in popliteal and brachial artery blood flow. MAP and AT were the largest independent determinants of lower and upper body partial occlusion pressures but greater variance was explained by regression models composed of DBP and BMI (48 %), and arm AT and DBP (30 %), respectively. In contrast, limb circumference has limited impact on the cuff pressure required for partial blood flow restriction. However, this may be due to the narrow range in limb circumference observed in healthy subject populations as investigated here. Nevertheless, the majority of the variance in partial occlusion pressure remains unexplained by the predictor variables assessed in the present study. Further investigation of the influence of local vessel characteristics is required if studies are to achieve an equal BFR between subjects. Sex sensitivities in the regulation of arterial vascular tone may also need to be considered.

## Abbreviations

AT: Adipose thickness
ANOVA: Analysis of variance
BFR: Blood flow restriction
BMI: Body mass index
CSA: Cross-sectional area
DBP: Diastolic blood pressure
EDHF: Endothelial-derived hyperpolarising factor
MAP: Mean arterial pressure
SBP: Systolic blood pressure
SEE: Standard error of estimates
TAMV: Time averaged mean velocity
